# Cardiovascular risk score in Rheumatoid Arthritis

**DOI:** 10.12669/pjms.323.9779

**Published:** 2016

**Authors:** Abrar Ahmed Wagan, Tafazzul E Haque Mahmud, Aflak Rasheed, Zafar Ali Zafar, Ata ur Rehman, Amjad Ali

**Affiliations:** 1 Dr. Abrar Ahmed Wagan, FCPS (Medicine). Department of Rheumatology, Sheikh Zayed Federal Post Graduate Institute, Lahore, Pakistan; 2 Dr. Tafazzul E Haque Mahmud, MRCP, FRCP. Department of Rheumatology, Sheikh Zayed Federal Post Graduate Institute, Lahore, Pakistan; 3 Dr. Aflak Rasheed, FCPS (Medicine) FCPS(Rheumatology). Department of Rheumatology, Sheikh Zayed Federal Post Graduate Institute, Lahore, Pakistan; 4 Dr. Zafar Ali Zafar, FCPS (Medicine). Department of Rheumatology, Sheikh Zayed Federal Post Graduate Institute, Lahore, Pakistan; 5 Dr. Ata Ur Rehman, FCPS (Medicine). Department of Rheumatology, Sheikh Zayed Federal Post Graduate Institute, Lahore, Pakistan; 6 Dr. Amjad Ali, MBBS. Department of Rheumatology, Sheikh Zayed Federal Post Graduate Institute, Lahore, Pakistan

**Keywords:** CVD risk score, Framingham risk calculator, Rheumatoid Arthritis, QRISK-2 calculator, **RA:** Rheumatoid Arthritis, **CVD:** Cardiovascular diseases, **DM:** Diabetes Mellitus, **CKD:** Chronic kidney diseases, **AF:** Atrial Fibrillation, **BMI:** Body Mass Index, **CHOL:** Cholesterol, **HDL:** High Density Lipoprotein

## Abstract

**Objective:**

To determine the 10-year Cardiovascular risk score with QRISK-2 and Framingham risk calculators in Rheumatoid Arthritis and Non Rheumatoid Arthritis subjects and asses the usefulness of QRISK-2 and Framingham calculators in both groups.

**Methods:**

During the study 106 RA and 106 Non RA patients age and sex matched participants were enrolled from outpatient department. Demographic data and questions regarding other study parameters were noted. After 14 hours of fasting 5 ml of venous blood was drawn for Cholesterol and HDL levels, laboratory tests were performed on COBAS c III (ROCHE). QRISK-2 and Framingham risk calculators were used to get individual 10-year CVD risk score.

**Results:**

In this study the mean age of RA group was (45.1±9.5) for Non RA group (43.7±8.2), with female gender as common. The mean predicted 10-year score with QRISK-2 calculator in RA group (14.2±17.1%) and Non RA group was (13.2±19.0%) with (p-value 0.122). The 10-year score with Framingham risk score in RA group was (12.9±10.4%) and Non RA group was (8.9±8.7%) with (p-value 0.001). In RA group QRISK-2 (24.5%) and FRS (31.1%) cases with predicted score were in higher risk category. The maximum agreement scores between both calculators was observed in both groups (Kappa = 0.618 RA Group; Kappa = 0.671 Non RA Group).

**Conclusion:**

QRISK-2 calculator is more appropriate as it takes RA, ethnicity, CKD, and Atrial fibrillation as factors in risk assessment score.

## INTRODUCTION

Rheumatoid arthritis is the most common inflammatory arthritis affecting 0.5 to 1% of general population worldwide.^1^ Several studies have documented increased morbidity and mortality in RA, in a Canadian study it was found that RA patients had a median survival 17 years shorter than expected for general population.^2^ Despite remarkable improvements in RA treatment, there is evidence indicating that the mortality gap between patients with this disease and the general population is not closing. Ischemic heart disease and heart failure now represent one of the most common causes of death in RA. Indeed, RA appears to represent an independent risk factor for ischemic heart disease, similar to diabetes mellitus.^3^ The rates of undiagnosed hypertension, elevated LDL, and type II diabetes are high in patients with RA and treatment target goals, as defined by current therapeutic guidelines, are achieved in only (40%)of RA patients with hypertension, (57%) of patients with elevated LDL and (57%) of patients with diabetes.^4^ Atherosclerosis (AT) was once considered to be a degenerative disease that was an inevitable consequence of aging. However, research in the last three decades has shown that AT is not degenerative or inevitable. It is an autoimmune-inflammatory disease associated with infectious and inflammatory factors characterized by lipoprotein metabolism alteration that leads to immune system activation with the consequent proliferation of smooth muscle cells, narrowing arteries, and atheroma formation. Both humoral and cellular immune mechanisms have been proposed to participate in the onset and progression of AT.^5^

Atherosclerosis is essentially an inflammatory disease, with levels of different biomarkers of inflammation such as C-reactive protein (CRP), interleukin-6, and N-terminal pro-hormone B-type natriuretic peptide (NTproBNP) correlating closely with subsequent cardiac events. In patients with rheumatoid arthritis, inflammatory markers, disease severity, and rheumatoid factor (RF) sero-positivity, seem to be associated with the risk of later development of atherosclerosis. Other non-traditional risk factors may also contribute to cardiovascular risk, and include the long-term effects of non-steroidal anti-inflammatory drug (NSAID) treatment, and the use of cyclo-oxygenase-2 (COX-2) inhibitors, corticosteroids and lack of physical activity and increased incidence of metabolic syndrome in RA.^6^-^8^ Systemic inflammatory response in RA is critical to accelerated atherogenesis operating via accentuation of established and novel risk pathways and long term suppression of systemic inflammatory response should be effective in reducing the risk of coronary heart disease.^9^

Benefits of estimating total CVD risk and treating accordingly are two fold, first is the direction of preventive efforts towards those who are likely to benefit and second is the reassurance and avoidance of side effects of medication in lower risk persons with economic implications.^10^

There is lack of consensus regarding CVD risk estimation in inflammatory arthritis some of well-known risk calculators either underscores or over estimates the risk and most of the guidelines on CVD prevention recommend that pharmacologic and non-pharmacological measures are based on person’s total CVD risk.

This proposed study under took two risk calculators one which is well known risk calculator Framingham risk calculator and other is new risk calculator called Qrisk-2 risk calculator with aim of CVD risk score estimation in RA and also to assess the usefulness of both.

## METHODS

This cross sectional comparative study was conducted in Department of Rheumatology, Sheikh Zayed Federal Post Graduate Medical Institute Lahore from April 2014 to April 2015. Sample size of 106 for each group with 95% confidence level and 90% power of test with expected odd ratio of CVD among R.A patients 2.5 as compared to non RA and 30% exposure to comparative group. Approval from Institutional Review Board and ethical committee was taken. Age and sex match study participants having diagnosis of RA and Non RA were enrolled from outpatient department. Written and informed consent were taken from each participant. RA was diagnosed according to 2010 American College of Rheumatology criteria.

### Exclusion Criteria

It was ascertained that all study participants had no CVD event (myocardial infarction, coronary artery disease/reperfusion therapy, stroke, transient ischemic attack) in past. Diagnosis of sero-negative RA and other connective tissue disorder like, SLE, Systemic sclerosis, overlap syndromes (SLE/RA, RA/Scleroderma, RA/ Polymyositis), osteoarthritis, psoriatic arthritis, use of biological DMARD’s, history of active malignancy, were taken as exclusion criteria.

Demographic data, pulse rate rhythm and questions regarding RA duration, cigarette smoking was asked in detail. Body Mass Index was calculated by dividing weight (in kilogram) by square of height (in meters). For every individual their resting blood pressure was measured with mercury sphygmomanometer for three times in sitting position and the mean of two was used for analysis and anti- hypertension medication use was noted.

Diabetes mellitus was defined by checking fasting blood glucose level on glucometer and FBG of >126mg/dl was considered abnormal, self-reported previous diagnosis of Diabetes Mellitus, current use of insulin and/ or oral hypoglycemic medications. Family history of angina and myocardial infarction in first degree relatives (father, mother, brother, sisters, grandparents), Atrial Fibrillation was diagnosed on the basis of irregularity in pulse rhythm on clinical examination and confirmed on electrocardiography. Chronic kidney disease staging was done on the basis of Cockcroft and gault method and only CKD 4 (GFR of 15 to 29 mL/min/1.73m^2^) and CKD 5 (GFR less than 15 mL/min/1.73m^2^) were used for risk score calculation as per risk calculator requirement.

Biological tests were performed after 12 hours of overnight fasting. With aseptic technique 10ml of venous blood was obtained. Samples were collected in BD (Becton, Dickinson and company) vaccutainer sample bottles (red-topped). All samples were on analyzed on Cobas CIII [ROCHE] through photo spectrometry for Serum Cholestrol, HDL, Serum urea and Creatinine levels.

After data collection, data was set in QRISK-2 calculator; 10-years risk score was calculated for every individual while in FRS score RA patient’s actual score was multiplied by factor of 1.5 as per recommendation. SPSS 22 was used to analyze the data, frequencies and percentages were calculated for categorical variables whereas mean with standard deviation and median with interquartile range were calculated for continuous variables. Normality of the data was checked by Shapiro Wilk test. Mann Whitney U test was used to compare the continuous data between RA and Non RA groups. Chi square and Fisher’s Exact test were used to compare the categorical data between RA and Non RA groups. Cohen’s Kappa statistics was used to determine the agreement between both risk calculators. A p-value ≤ 0.05 was taken as significant.

## RESULTS

This study shows the female gender was predominantly more in number 86 (81.1%) in RA and Non RA groups, while male gender comprised of 20 (18.9) with (p-value > 0.999). In RA group was significantly more number of cases with positive family history of Myocardial infarction were detected 38 (35.8%), than Non RA Group-16 (15.1%) with (p-value 0.001).

Non RA group showed more number of cases that were labeled as chronic kidney disease 11 (10.4%) than RA Group-II (1.9%) with (p-value 0.010) Table-I. In this study mean age of RA group was (45.1±9.5) and Non RA group was (43.7±8.2) with (p-value 0.332).

**Table-I:** Comparison of risk factors for cardiovascular disease between RA and Non RA groups.

	*RA Group n (%)*	*Non-RA Group n (%)*	p-value*
*Gender*			
Male	20 (18.9)	20 (18.5)	
Female	86 (81.1)	86 (81.1)	> 0.999
*Hypertension Medication*			
No	82 (77.4)	70 (67.0)	
Yes	24 (22.6)	35 (33.0)	0.092
*Smoking Status*			
No	90 (84.9)	74 (84.0)	0.850
Yes	16 (15.1)	17 (16.0)	
*Diabetes Mellitus*			
No	88 (83.0)	80 (76.4)	0.232
Yes	18 (17.0)	25 (23.6)	
*History of M.I in Family members*			
No	68 (64.2)	80 (84.9)	0.001
Yes	38 (35.8)	16 (15.1)	
*Chronic Kidney Disease*			
No	104 (98.1)	97 (89.6)	0.010
Yes	2 (1.9)	11 (10.4)	
*Atrial Fibrillation*			
No	104 (98.1)	102 (96.2)	0.683
Yes	2 (1.9)	4 (3.8)
*Framingham score*			
<10%	61 (57.5)	73 (68.9)	0.011
10 – 20	12 (11.3)	18 (17.0)	
>20	33 (31.1)	15 (14.2)	
*QRISK-2 Risk score*			
<10%	62 (58.5)	71 (67.0)	0.435
10 - 20%	18 (17.0)	15 (14.2)	
>20%	26 (24.5)	20 (18.9)	

* Chi-square test / Fisher’s exact test.

There were statistically significant number of cases with higher BMI in RA group with mean BMI score of (28.7±5.9) than Non RA group (26.1±3.3) with (p-value 0.001) and RA group cases were having higher mean Cholesterol levels (187.1±35.9) than Non RA cases (179.1±31.9) with (p-value 0.117).

The mean estimated risk score with Qrisk-2 calculator in RA group was (14.2±17.1) and in Non RA group (13.2±19.0) which was statistical insignificant difference p-value 0.122. The mean estimated score with Framingham risk score in RA group was (12.9±10.4) and Non RA group (8.9±8.7) with statistical significant difference (p-value 0.001) Table-II.

**Table-II:** Comparison of age, BMI, systolic blood pressure, cholesterol, HDL level, Framingham score and 10-years risk score between RA and non-RA Group.

	*RA Group*		*Non- RA Group*		

	Mean±S.D	Median (IQR)	Mean±S.D	Median (IQR)	p-value*
Age	45.1±9.5	46 (37.8 – 52.3)	43.7±8.2	45 (37 – 50)	0.332
BMI	28.7±5.9	27.9 (23.7 – 31.7)	26.1±3.3	26.0 (24.0 – 28.0)	0.001
Systolic blood pressure	127.2±16.6	124 (116 – 140)	125.1±10.6	122 (120 – 130)	0.599
Cholesterol level	187.1±35.9	180 (158.7- 202.5)	179.1±31.9	170 (156.0- 190.0)	0.117
HDL level	44.5±6.9	42 (40 – 50)	46.5±6.9	45 (40 – 50)	0.016
Framingham score	12.9±10.4	8.1 (4.0 – 23.9)	8.9±8.7	5.3 (2.4 – 12.1)	0.001
QRISK-2 score	14.2±17.1	6.1 (2.3 – 20.2)	13.2±19.0	5.1 (1.2 – 16.1)	0.122

* Mann-Whitney Test.

**Fig.1:**
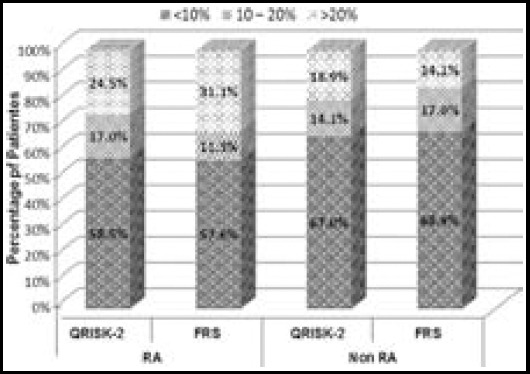
The estimated 10-years cardiovascular risk score according to Framingham and QRISK-2 in RA and Non RA groups.

Both calculators predicted the nearly same number of cases in mild and moderate risk category. In RA group QRISK-2 (24.5 %) and FRS (31.1%) cases were in higher risk category (Fig.1).

Cohen’s Kappa statistics was used to determine the agreement between both risk calculators. The maximum agreement scores between both calculators was observed in both groups (Kappa = 0.618 RA Group; Kappa = 0.671 Non RA Group) with p-value < 0.001) Table-III.

**Table-III:** Comparison of Qrisk-2 risk score and Framingham risk score for cardiovascular disease in RA and Non RA group.

	*Risk percentage*	*QRISK-2 score n (%)*	*Framingham score n (%)*	*Kappa value*	p-value*
RA Group	<10%	62 (58.5)	61 (57.5)	0.618	< 0.001
	10 – 20	18 (17.0)	12 (11.3)		
	> 20	26 (24.5)	33 (31.1)		
Non-RA Group	<10%	71 (67.0)	73 (68.9)	0.671	< 0.001
	10 – 20	15 (14.2)	18 (17.0)		
	>20	20 (18.9)	15 (14.2)		

* Cohen’s Kappa Test.

## DISCUSSION

Rheumatoid arthritis as chronic inflammatory joint disease is not limited to synovium, but cardiovascular involvement is the major concern now a day along with joint problems. Cynthia S Crownson et al, in her study reported that majority of RA patients with angina remained undiagnosed, myocardial infarction patients were less able to receive reperfusion therapy and there is lower prevalence of primary & secondary prevention regarding cardiovascular diseases.^11^

Crowson et al, in her study used General Framingham, Office based Framingham and Reynolds score system to predict the 10-year risk of CVD, and found these underestimated the risk in certain age groups and for others it overestimated. In USA and Europe, General, Office based Framingham, Reynolds score and Systemic coronary risk evaluation (SCORE) models are often used, but all of these don’t account RA as independent risk factor and accuracy is debatable.^12^

Score model is optimized for European clinical practice, Swiss society of cardiology recommended that absolute CVD risk score subsequently requires the multiplication by factor of 1.5, while EULAR contrary to above has advised the multiplication factor use when RA disease more than 10-years duration, RF or ACPA positivity, presence of certain extra articular features.^13^,^14^ Recently NICE has validated the QRISK-2 risk calculator for cardiovascular risk screening.^15^,^16^

Hilal Maradit et al. in their retrospective inception cohort study of RA and Non RA cohort noted more than half of RA cohort with mean age of 56.8 years, had >10% risk, within 10-years of RA onset, and age played very significant role in increasing the CVD risk.^17^ Mike JL Peters et al. reported that risk of CVD in RA was more than the general population and comparable with magnitude of risk posed by type 2 diabetes mellitus.^18^

Inmaculada del Rincon et al, reported that RA and established CV risk factors, contribute significantly to carotid atherosclerosis and factors may modify one another’s effects, but non-traditional risk factors also play a significant role in CVD events.^19^

Daniel H, Solomon and Inmaculada del Rincon et al, in their studies found increased level of cholesterol higher level of BMI and patients with positive history of Myocardial infarction in close family members.^20^

In our study we found higher cholesterol levels, higher BMI levels and more cases with positive history of myocardial infarction in close family members in RA group. With disease duration of less than a decade, almost (20-30%) of total RA group patients were falling in higher risk category which signifies indirectly that these patients are having increased risk of developing CVD events than general population and requires intervention in terms of life style modification and pharmacotherapy.

Qrisk-2 is appropriate as it takes ethnicity and RA as factor in risk estimation and gives precisely exact risk score beyond 30% which is not the case with other calculator and FRS requires multiplication by factor of 1.5 when it is used in Rheumatoid Arthritis.

### Limitations

Only logical method to assess the accurate predictive capability of different risk assessment tools is large scale, long term prospective study.

## CONCLUSION

Rheumatoid arthritis patient’s needs to be encouraged to avoid sedentary life style, use healthy diet, remain physically active as much possible, minimize illicit drug use quit smoking and cardiovascular disease risk estimation should be done by regularly through validated risk calculators.
